# Closing investigations: The role of national policy in shaping structural, organisational and relational constraints on learning from patient safety incidents

**DOI:** 10.1016/j.ssci.2025.106999

**Published:** 2025-12

**Authors:** Polina Mesinioti, Carl Macrae, Laura Sheard, Sarah Hampton, Gemma Louch, Jane O’Hara

**Affiliations:** a Department of Health Sciences, Seebohm Rowntree Building, University of York Heslington, York YO10 5DD, UK; b Nottingham University Business School, Wollaton Rd, Lenton, Nottingham NG8 1BB, UK; c School of Health Sciences, University of Manchester, Oxford Road, Manchester M13 9P, UK; d Clinical, Educational and Health Psychology, University College London, 27 Gordon Sq, London WC1H, UK; e School of Healthcare, Baines Wing School of Healthcare, University of Leeds, 13 Beech Grove Terrace, Woodhouse, Leeds LS2 9DA, UK; f THIS Institute, University of Cambridge Strangeways Research Laboratory, 2 Worts’ Causeway, Cambridge CB1 8RN, UK

## Abstract

This study aimed to understand the role of a national patient safety policy, the Serious Incident Framework, on local organisational practices of responding to, investigating, and learning from patient safety incidents in the National Health Service (NHS) in England. Qualitative interviews were conducted with healthcare professionals in six NHS organisations and analysed using inductive thematic analysis and taking a constant comparison approach. Systemic challenges linked to the policy’s prescriptive requirements were identified, including its emphasis on metrics such as incident closure and harm levels, which often obscured meaningful learning and systemic improvement. The findings highlight the misalignment between the policy’s key aims and principles and its practical implementation, revealing an ‘industry of investigations’ that risked turning the investigative process into a compliance-oriented ‘tick box exercise’. Furthermore, the overspecification of performance requirements coupled with the underspecification of substantive guidance led to variability in investigative processes, organisational capacity and resources, and investigator training and expertise. The involvement of patients and families affected by safety incidents was found to be inconsistent and often limited, with perceptions of senior managers and frontline staff underlining some tensions in operationalising large patient safety policies. The analysis considers how the development and implementation of national safety incident policies needs to carefully and intelligently balance the need for adaptive flexibility, clarity of guidance, and specification of organisational resourcing and infrastructure to ensure future national policy can effectively support local practices of learning from safety incidents.

## Highlights

• National patient safety policy can distort local efforts to learn from incidents.
• Prescriptive metrics for investigation processes can preclude learning.
• Investigative structures and resources shape capabilities for learning.
• Compliance pressures can crowd out collaborative capabilities.

## 1 Introduction

Investigating and learning from serious safety incidents remains one of the foundational activities of safety management and is a key policy priority across a range of safety–critical sectors. How such policies translate into organisational practice and shape investigative activity is therefore a critical question. This is particularly the case in healthcare, where large numbers of serious safety events occur, and where a wide range of policies, standards and guidelines have been produced in an effort to standardise and support effective incident investigation and learning. Patient safety remains a major challenge in health systems around the world (World Health Organization (WHO), 2019), with around 1 in 10 patients being harmed, more than 3 million deaths occurring annually due to unsafe care (World Health Organization (WHO), 2023), and around 1 in 20 patients experiencing some form of preventable harm across care settings (Panagioti et al., 2019). Patient safety incidents can have devastating consequences for patients and their families and pose a significant financial burden on healthcare systems worldwide. More than 12 % of national healthcare costs are accounted for by the clinical impact of unsafe care (Slawomirski & Klazinga, 2022).

In light of this, over the past two decades considerable policy attention and organisational effort has focused on establishing and improving the way patient safety incidents are investigated and learnt from. In health systems around the world, the reporting and analysis of safety incidents has become a central strategy of patient safety management (Macrae, 2014, Wami et al., 2016). In England, for instance, more than 2 million patient safety events are now reported annually across the English National Health Service (NHS) and are collated in one of the world’s largest safety incident reporting programmes (NHS, 2022). Similarly, since 2010, England has established a national standardised policy that mandates and specifies how NHS organisations should respond to and manage the most serious incidents, to support learning and safety improvement (National Patient Safety Agency, 2010). This ‘Serious Incident Framework’ policy (‘SIF’ hereafter) was in place for over a decade and set out national requirements including defined thresholds of serious incidents requiring investigation, the required processes and timelines for investigation and reporting of those incidents, and the roles and responsibilities of those involved.

Despite the effort that was put into implementing this national policy, and the espoused aim of the policy being to support learning and improvement (the full title of the 2015 edition of the policy was “Serious Incident Framework: Supporting Learning to Prevent Recurrence”, NHS England, 2015), it has been generally acknowledged in both academic literature and national inquiries that for the duration of this policy being in place, investigation and learning across the NHS continued to fall short of expectations. Many of the organisational systems, practices and skills that support learning remained underdeveloped (Louch et al., 2025, Wood et al., 2023) and failures to effectively manage and learn from safety incidents continued to be identified as key contributors to major healthcare disasters (Hughes, 2023, Kirkup, 2015). Some of the challenges identified include ineffective incident follow up (Archer et al., 2020), a pervasive fear amongst staff of being inappropriately blamed in response to safety events (HSIB, 2021, Tasker et al., 2023), limited expertise in safety investigation and systems-oriented approaches to safety analysis (Adamson et al., 2022, Wood et al., 2023), poor engagement of staff and families in investigation processes (CQC, 2016), and responses to incidents overly focused on individualised issues rather than seeking out opportunities to improve systems and wider practices (HCI, 2021, Bakhbakhi et al., 2019).

Accordingly, the puzzle that this paper explores is: what is the role of national policy in either supporting, or constraining, efforts to learn effectively from serious safety incidents? And how can safety policy that clearly espouses a central aim of supporting learning and that is widely complied with and robustly governed, persistently fail to achieve that aim? These questions represent a wider set of challenges relating to policy implementation. Policy instruments necessarily privilege, legitimise and encourage certain types of activities and organisational routines, but these are not always the activities and behaviours that the policy is explicitly intended to create (Baldwin et al., 2013). Indeed, policies and related regulatory and compliance activities that foreground and focus on readily measurable proxy indicators, or easily audited targets, can drive a range of unintended, unexpected and perverse organisational consequences (Power, 1999, Hayes et al., 2023). These complications, and the unintended consequences of safety policy on organisational practice, are particularly well-illustrated in the arena of healthcare safety investigation, as evidenced by the conflicting policy aspirations and practical realities of the NHS SIF.

The present study explores these issues through an in-depth qualitative interview study with 49 NHS staff involved in managing serious incidents. The study specifically aims to understand how the national SIF policy shaped and constrained local practices of safety investigation and learning in NHS organisations, prior to this policy being phased out in 2023. In the following sections, the SIF policy and its role in the English NHS is first introduced in detail. Then, the research approach and methods are explained, detailing the interviews that were conducted and the qualitative analysis of the resulting data. The findings are then presented, which explore the structural, organisational and relational constraints on learning that emerged from the implementation of this national policy. Finally, the implications are considered particularly in relation to the policy’s role in enabling safety incident management, investigation and learning.

## 2 National policy for managing serious patient safety incidents in England

The English NHS has, since 2001, been subject to a range of national programmes, initiatives and policies that have been directly targeted at improving patient safety (Department of Health, 2000). One of the major areas of focus has been on developing national, standardised approaches to reporting, managing and responding to safety incidents. The SIF, published in 2010 and updated in 2015, was the first national policy to set out requirements for the management and investigation of serious incidents by NHS Trusts (organisations providing care) across England. The policy described ‘Serious Incidents’ (or ‘SIs’) as “events in health care where the potential for learning is so great, or the consequences to patients, families and carers, staff or organisations are so significant, that they warrant using additional resources to mount a comprehensive response” (NHS England, 2015, p. 12). The specific definition and criteria for whether an incident is regarded as an ‘SI’, and therefore worthy of investigation, run to almost two pages, but focus on harmful or damaging outcomes: primarily acts or omissions that result in “unexpected or avoidable death”, “unexpected or avoidable injury”, “actual or alleged abuse”, and any incident that “prevents, or threatens to prevent, the organisation’s ability to continue to deliver an acceptable quality of care” (NHS England, 2015, p. 13).

A set of seven key principles was proposed as underpinning the management of all SIs (NHS England, 2015):

1. **Open and transparent**: Investigations must prioritise the needs of those affected, including patients, families and staff, through openness, honesty, early apologies and ongoing communication.
2. **Preventative**: The goal of investigations is to learn and prevent future harm by identifying system and process weaknesses and not to assign blame or determine legal responsibility.
3. **Objective**: Investigations must be conducted independently of those involved in the care in question, to ensure impartiality and avoid bias arising in team or organisational cultures.
4. **Timely and responsive**: Serious incidents must be reported without delay and managed appropriately with responsiveness to the unique circumstances of each case.
5. **Systems-based**: Investigations must use a systems-based approach, typically Root Cause Analysis (RCA), understand what happened, how, and why and considering environmental and human factors.
6. **Proportionate**: Investigations should be scaled appropriately to the severity and complexity of the incident, considered on a case-by-case basis ensuring resources are focused appropriately.
7. **Collaborative**: Organisations must work in partnership when incident management requires coordination across multiple organisations, with clearly defined roles and shared procedures.

**Fig. 1:**
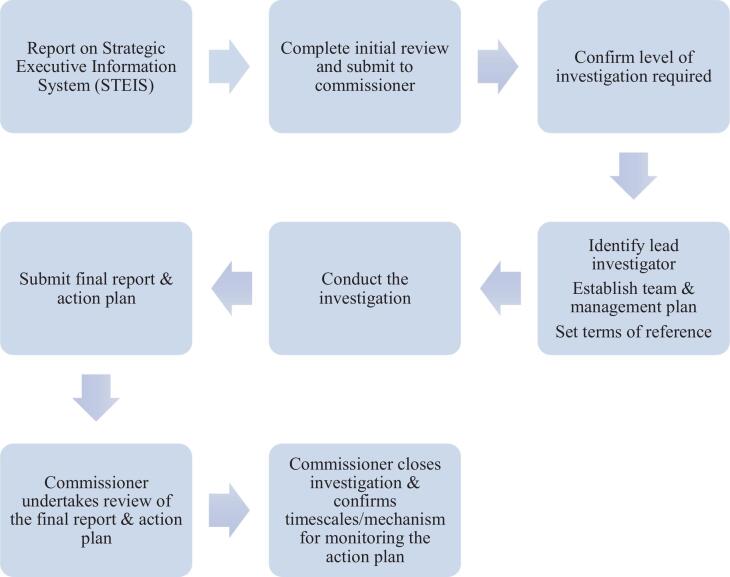
The Serious Incident Management Process.

A key element of the SIF was accountability and monitoring, with the framework detailing that all SIs must be reported by the NHS Trust in which they occurred to the body that commissioned the care, with these ‘commissioners’ responsible for reviewing the investigation, ‘closing’ the investigation once they are satisfied it has been completed, and subsequently monitoring progress against any resulting action plans. The ‘Serious Incident Management Process’ described by the SIF is briefly summarised in Fig. 1. The NHS organisation (known as the ‘provider’) is responsible for establishing an appropriate investigation team, and SIF indicated the essential competencies required of all members of the investigation team as including: knowledge of the systems investigation process and the skills to deliver it; effective report writing; experience in facilitating patient and family engagement; understanding of the clinical specialty involved; and appropriate mechanisms to share lessons locally and nationally.

The NHS organisation is required to determine the ‘level’ of investigation that is necessary, after an initial review of the incident; though this could be altered as new evidence emerged. There were three levels of systems-based investigations, summarised in Table 1. Each level of investigation had a required timeline for completion, ranging from 60 working days to 6 months. An investigation report and an action plan had to be produced, and the report was expected to identify ‘root causes’ and ensure that the conclusions were justified and based on relevant evidence, and that recommendations were feasible to implement. The policy detailed the minimum requirements of an action plan, including that each recommendation be linked to a clearly articulated action and a clear deadline for its completion. These actions, in turn, should aim to reduce the risk of the incident’s recurrence, and address systemic weaknesses. The SIF, with its highly centralised and standardised approach, was phased out in 2023, and replaced by a new approach – the ‘Patient Safety Incident Response Framework’ (‘PSIRF’) that is designed to give NHS organisations more localised control over what, and how, to investigate (NHS England, 2024).

**Table 1:** Levels of investigations defined by the SIF (information taken from NHS England, 2015).

Level of investigation	Type of incident	Timeline
Level 1	Less complex incidents managed by small teams at a local level	Within 60 working days of the incident being reported
*Concise internal investigation*		
Level 2	Complex incidents managed by a multidisciplinary team involving experts and/or specialist investigators	
*Comprehensive internal investigation*		
Level 3	Incidents in which the integrity and/or objectivity of the investigation might be challenged	Within 6 months of the investigation’s commission
*Independent investigation*		

## 3 Research methods and approach

This study aimed to understand the role of a national patient safety policy, the SIF, on local organisational practices of responding to, investigating, and learning from patient safety incidents in the NHS. It particularly aimed to explore how the SIF was experienced by the healthcare professionals who were responsible for managing serious incidents under this policy. This study is part of a larger research programme – the *Response Study* – that is studying, in real-time, the 2023 implementation of NHS England’s new approach to safety investigation.^1^ Accordingly, the study reported here aimed to understand the policy context and organisational practices, as they existed prior to this change in 2023.

### 3.1 Data collection

One-off semi-structured interviews were conducted with NHS staff from six Trusts (*n* = 49) between July 2023 and April 2024. Expressions of interest were sought for NHS Trusts to participate in the wider Response Study programme. Following this, three mental health Trusts and three acute Trusts were selected from three NHS England and Improvement (NHSEI) regions to reflect variation in organisation size, diversity of local population and staff demographic information. These contextual factors were considered based on earlier work illustrating that organisational factors and population demographics, such as ethnicity, impact serious incident management in the NHS (Farrant et al., 2022). To illustrate the volume of patient safety incidents handled, while not all Trusts publish their figures annually, available data from selected Trusts indicate that each reported between 8,000 and 19,000 incidents in 2022–23 (exact figures are withheld to maintain anonymity). This variability in incident volumes was considered potentially relevant to how incidents are managed, given that higher numbers may create additional pressure on organisational resources.

The interviews were conducted by PM and SH via Microsoft Teams and were video recorded. None of the researchers involved in this study have formal or professional ties to NHS England. This ensures that the research is conducted impartially, thus preserving its objectivity and credibility. The interviews lasted between 25–40 min. The topic guide broadly sought to understand the management and processing of patient safety incidents across Trusts under the SIF. Questions aimed to capture the ‘who’, ‘what’ and ‘how’ of investigations, including personnel, processes and procedures. We specifically asked about priorities, decision making, responsibilities, the fairness and equality of SIF processes, challenges, opportunities, and potential changes resulting from safety reporting and investigations.

We recruited participants from different professional groups to gain a holistic understanding of approaches and practices for managing patient safety incidents and how these varied within and across Trusts. We targeted clinical, patient safety, senior management, governance, and leadership teams. Some of the involved roles were patient safety managers, clinical governance managers, senior clinical nurses, and executive medical directors. We interviewed between 5 and 10 participants per Trust, totalling 49 participants. The distribution of participants is summarised in Table 2. The interviews were conducted during the period of transition, as the NHS moved from the previous SIF policy to a new approach that was published in 2023, providing participants with the opportunity to reflect on past experiences of managing incidents under the SIF policy. The study received ethical approval from London-Stanmore Research Ethics Committee (22/PR/1600).

**Table 2:** Participants recruited per Trust.

	Trust 1	Trust 2	Trust 3	Trust 4	Trust 5	Trust 6	Total
Participants from patient safety teams	2	4	1	4	3	1	15
Participants from clinical teams	2	4	6	−	3	1	16
Participants from senior management, governance, and risk management teams	5	2	2	2	4	3	19
Total	9	10	9	6	10	5	49

### 3.2 Data analysis

An inductive thematic approach was employed, whereby the analysis was not based on any preexisting theoretical framework; rather, the identified patterns emerged directly from the data. We took a constant comparative approach (Charmaz, 2006, Fram, 2013), focusing on core similarities and differences between the Trusts. This entailed analysing the interviews from each Trust separately and devising themes and subthemes, before comparing them to those of the Trusts previously analysed. Our analytical process involved: data collection, familiarisation with the dataset, initial coding of interesting features, building that initial coding up to broader themes consistent across interviews, refinement of the themes in each Trust, comparing the themes of each Trust with the previous ones, reaching consensus on overall themes across Trusts, and writing. The key distinction of our inductive, ‘bottom-up’ approach from deductive approaches is that the generated themes are data-driven rather than theory-driven; this allowed us to capture the nuances of the dataset, rather than testing whether something we predicted is there. PM led the analysis with regular discussions with LS and the wider team. All authors read drafts of the analysis and provided feedback.

## 4 Findings: The structural, organisational and relational impacts of national safety incident management policy

The SIF was founded on a set of principles that emphasised proportional, collaborative, and contextually sensitive approaches to investigation. However, this analysis indicates that, in its design and implementation, the SIF created a set of interlocking constraints into the work of serious incident investigations. First, it created structural constraints by imposing a rigid temporal architecture organised around fixed reporting timelines, as well as a highly stochastic logic that tied investigative action and resource allocation to the severity of harmful outcomes, which necessarily occur irregularly and unpredictably. Second, these policy design choices shaped and constrained the organisation of investigative activity and capacity. Investigative capacity and resources were often mismatched to the investigative requirements set out in the SIF. These capacity constraints, in turn, impacted the depth and quality of interaction and engagement with stakeholders, particularly patients and families. Third, the SIF configured investigative roles and relations in ways that made genuine learning challenging. It placed heavy reliance on individual competence, which was often variable and not reliably supported by training, meaning investigative approach and quality was influenced by these individual variabilities. Further, role relations, and the connections with teams and between leaders and other stakeholders, were challenging to build effectively, particularly when attention was focused on governance and administrative processes. These constraints created by the SIF are explored and elaborated in turn below.

### 4.1 The structuring of investigative processes

Despite the SIF’s stated aims of supporting learning and prevention, and its espoused principles such as investigations being proportional, collaborative and responsive to unique circumstances, two foundational aspects of the SIF imposed strong structural constraints on how incidents were managed and responded to within Trusts. First, the SIF imposed a strong temporal structure through a set of required timelines. Second, the SIF imposed an unpredictable stochastic structure by anchoring required investigation activities to the severity of an incident’s outcome.

#### 4.1.1 Temporal structure and reporting timelines

The SIF was highly prescriptive in terms of the timelines and reporting requirements that were mandated and had to be met at key points along the incident management and investigation process. These temporal prescriptions acted as one of the key control mechanisms instituted by the policy and established a strong temporal structure that was both highly prescriptive and heavily performance-managed by external parties, such as commissioners and regulators. These timelines were a defining feature of the policy, and included: 2 working days for NHS Trusts to inform commissioners and other relevant parties about an incident; 72-hours for NHS Trusts to review an incident and confirm whether it should be considered an SI according to the SIF definition, and determine the level of investigation required; 60 days to complete both concise and comprehensive investigations; and 6 months to organise and complete an independent investigation.

These reporting timelines created a strong temporal structure that was experienced as the central organising feature of incident management processes under the SIF policy. Despite the SIF’s aims, the common experience of participants was that the primary focus of effort was complying with the temporal requirements of the policy and marking stages as complete, or ‘green’:

And I think largely when it’s been reported about actions from SI’s, it’s been more about ‘this is over the time frame’ and the focus has been ‘**it needs to be green as opposed to anything else**’ as in green being that it’s completed. So we’re very much focused on that. How many you’ve opened? How many have been closed, how many are overdue? […] It’s just process orientated rather than anything else. [Trust 4, Interviewee 3, Patient Safety Team]

Participants emphasised that the consequences of the mandated 60-day timeframe to complete investigation was that the process could be experienced as a tick-box exercise rather than one that was oriented to fostering genuine change and improvement, resulting in sometimes limited opportunities for effective patient and family involvement. The pressures were felt particularly acutely due to the attention given to meeting these timelines by external actors, including commissioners and England’s health and care regulators, the Care Quality Commission (CQC):

You've got to get your report in within 60 days. There's no movability on that. And if you don't get it in by that time frame, it's considered overdue […] **it's frowned upon then when you have your CQC ratings as well.** You have your CQC visits and then they're saying, ‘oh, well, actually, you've got a number of investigations that aren't completed on time’. I'm not sure how meaningful that did become for patients and families. It was like, We've got to get it done. We've got to get it done. I need to meet with you on this date and we've got to get this bit done. [Trust 2, Interviewee 8, Patient Safety Team]

The temporal structure created by the SIF reporting timelines, along with the associated external auditing, assessment and compliance activities that monitored and managed performance against those timelines, were experienced by participants as the central organising principles of the SIF. This focused organisational attention on the process of incident management, rather than the process of learning.

#### 4.1.2 Stochastic structure and harmful outcomes

The SIF was highly prescriptive regarding the criteria and thresholds for which incidents were determined to be SIs requiring investigation. As described previously, these criteria were focused on harmful or damaging outcomes. Incidents that resulted in patient death, serious injury or abuse, or that damaged the organisation’s ability to provide ongoing care, were the primary criteria for requiring an incident to be considered ‘serious’ and therefore requiring of an investigation. The ‘level’ of investigation was then determined by an assessment of the complexity of the incident and the investigation team that may be required. The investigative activities within NHS Trusts were therefore organised around, and structured according to, the stochastic and unpredictable occurrence of harmful adverse outcomes. Safety investigation, and associated learning activities, were not organised around strategic safety priorities or careful assessments of risk; they were driven by the occurrence of harm.

The consequences of this focus on harm were twofold. First, participants described how investigative activities and resources were necessarily applied to the repeated investigation of commonly occurring and well-understood events:

We reported 44 pressure ulcer SI’s in one year, which is 44 reports need to be doing and often the same five or six contributory factors, like pervasive, it runs through them all. […] you've got to do this report, so I think that maybe felt like a bit of a… just on that hamster wheel […] **Why would we need to do a report to tell us something that we already know, take people out of clinical practice to do that report?** [Trust 3, Interviewee 3, Governance Team]

The SIF structured the work of investigators in a way that focused attention and resources on events that resulted in harm and damage, irrespective of what might be learnt from those or whether that time and attention might be better focused on addressing the already understood risks in a particular area. Participants found that this focus on harm meant that more subtle indicators of risk, and other patterns worth of attention and investigation, would neither meet the criteria for investigation if they were noticed, and might in any case not be noticed given the priority and attention given to investigating events associated with harmful outcomes:

There was a lot of things that needed to be better [in the SIF criteria]. I think it was very prescriptive before, and I think because of that, we sometimes missed the subtle things that were going on. **Everybody uses falls and pressure ulcers, but they're just an easy one.** You might be seeing a real spike on a ward of falls. They might be getting suddenly in one month have had 30 falls. [Trust 5, Interviewee 3, Patient Safety Team]

By structuring investigation processes around harmful outcomes, the SIF was experienced by participants as creating a narrow and limited focus constrained by whichever events or types of incidents happened to result in harm, as well as requiring finite attentional and organisational resources to be focused on repetitive investigations rather than active improvement. This created a structure in which it was difficult to strategically identify and assess risks to patient safety or allocate resources to maximise the opportunities for learning and meaningful change. That is, the policy created organisational structures which ran counter to its initial aims.

### 4.2 The organisation of investigative activity and capacity

The SIF was ostensibly developed to support and encourage responses to serious incidents that actively and openly engaged with and supported “the needs of those affected” (NHS England, 2015, p. 21) by SIs, and that were oriented to preventatively identifying and addressing “weaknesses in a system and/or process… to prevent similar incidents occurring again” (ibid.). However, and because of the temporal and stochastic structure imposed on investigative work by the SIF, participants reported that there was often a significant mismatch between investigative resources and the requirements imposed by the SIF, and that these capacity constraints limited the quality and extent of coordination and engagement activities during investigations.

#### 4.2.1 Mismatch of requirements and resources

The investigative criteria and temporal requirements of the SIF, explored in Section 4.1, placed considerable pressure on organisations, and many participants reported a mismatch between the requirements to investigate and the organisational capacity to deliver those investigations. While participants in one Trust acknowledged they were unusual and ‘lucky’ to have an adequate team of investigators, most participants described a situation where the requirements to investigate outpaced the capacity to investigate:

[…] there are too many investigations for the number of substantive people we've got, even on the mental health side. But what you find is people are doing investigations alongside their job, which then they've got, you know, they're doing a full-time job anyway and then they've got to do an investigation. [Trust 2, Interviewee 1, Patient safety team]

These capacity constraints were exacerbated by the common approach of organising investigative work as, in many cases, an ‘add on’ to the usual work of staff. While the SIF was highly prescriptive regarding what types of events require an investigation, and the timelines for conducting and reporting those investigations, it provided very little indication of the organisational capacity, investigative resources or staff competencies and capabilities that should be in place to support investigative work. The mismatch of requirements and resources was also described by participants as having a critical impact on organisational capacity to follow up investigations with actions and to implement required changes and improvements:

That’s quite tricky for us in division to change whatever because we don’t have the resource, we don’t have the capacity. So, we went through a phase where, so women’s and children’s were the worst but because of the scrutiny around maternity, women’s and children’s always seemed to have the most RCAs and seemed to have the most actions, it ended up with a list of 20 actions, two thirds of which will be read and two thirds of them even sitting there. [Trust 6, Interviewee 3, Senior Management Team]

This mismatch was further amplified by the SIF investigative requirements which could result in large numbers of action plans being produced in response to the large numbers of serious incidents being investigated. This often led to large numbers of individual action items that challenged organisational capacity:

…Whereas our medicine division, huge division, multitude of specialties and it can sometimes be quite hard to get them to focus and feedback on individual actions related to a particular incident and also that can be due to capacity […] So their ability to then have downtime if you like to then ensure that actions are implemented has lessened. [Trust 4, Interviewee 2, Governance Team]

The large number of investigations each Trust was required to conduct, which led to a large quantity of individual incident-derived action plans, combined with a relatively small number of investigators and limited capacity within healthcare teams, created significant challenges and bottlenecks in efforts to carefully investigate and actively improve the safety of systems and processes.

#### 4.2.2 Capacity constraints on interaction and engagement

One of the key areas where capacity constraints impacted the conduct of incident investigations was in relation to the interaction and engagement with patients and families. This was reported by participants as being a key challenge, and one of the main areas of organisational activity that was diminished when capacity was limited, running counter to the original aim and aspiration of the SIF. Experiences differed, but participants commonly described a lack of meaningful engagement with patients and their families in the investigative process, reflecting that they would be informed that a patient safety incident had occurred, but would then typically only have a peripheral role in the investigation, often limited to receiving a copy of the final report, or being provided an opportunity to raise questions at late stages in the process:

It almost feels like **the patient and their families are a bit of not an afterthought, but they're very much not at the centre of the investigation**. It's more how the report looks, the effort that's gone into it. [Trust 4, Interviewee 2, Governance team]

Participants perceived this to be a significant omission and regretted that patients and their families were not always given the opportunity to speak to the investigators and share their experiences in meaningful ways. Moreover, several participants highlighted that patients and families might typically only receive a copy of the final report and an indication of the actions there were being planned in response, but were not subsequently informed of how those improvements had been made:

*Interviewer*: And would you say that the people affected by an incident are notified or involved in changes and improvements?

*Participant*: I would say probably not. When all the actions have been completed, they obviously get a report of what the actions will be. **That's probably something we need to get better at.** [Trust 1, Interviewee 9, Clinical team]

Despite these challenges, a commitment to involving patients and families throughout the process remained and some participants indicated that considerable efforts were made to include the patient and family perspective when the initial scope of an investigation was planned, and to meet with them at the end of the process to explain what was found and what will be done in response:

From the very outset, the patient is involved or the family is involved as part of the terms of reference setting. […] We make sure that their voice is captured there. They'll receive regular updates on the progress of their review. At the end, we will always meet with the patient or their family and take them through the review and how we will be implementing the recommendations for that. [Trust 1, Interviewee 8, Patient Safety team]

However, while the critical importance of engaging with and involving patients and families was acknowledged and emphasised, the capacity constraints that arose when organisations attempted to meet requirements to investigate large numbers of serious incidents typically tempered these aspirations. For the most part, participants indicated there was only ever limited capacity to genuinely and meaningfully engage with patients and families throughout an investigative process.

### 4.3 The construction of investigative roles and relations

Investigative work depends on the skills, competencies and capabilities of safety investigators and the organisational teams that surround and support them. The SIF was based on principles that specifically acknowledged that “investigation must be undertaken by those with appropriate skills, training and capacity” (NHS England, 2015, p. 23) and that “[t]here must be clear arrangements in place relating to the roles and responsibilities of those involved” (NHS England, 2015, p. 24). These principles were, however, undercut by the limited provision of information or requirements regarding the skills and competency of investigators within the SIF policy, as well as the tensions and challenges that existed in practice in organisations when it came to establishing persistent, robust and embedded safety roles and relations to support safety investigation processes.

#### 4.3.1 Competence reliance and variability

Investigative workloads were typically highly demanding, and the conduct of safety investigations and incident management was heavily dependent on the work of a relatively small number of investigators. The competence, style and approach taken by these individuals was described by participants as particularly important. While the SIF was clear in stating that investigative processes should be fair and treat staff consistently, even going so far as to state there should be “zero tolerance for inappropriate blame” (NHS England, 2015, p. 22), participants described how staff’s experience of investigation processes was highly dependent on the individual investigators leading the process:

Think it's very much about that clinical leadership and the culture, and the psychological safety that they bring on. And I think the vision of who’s implementing and who the team that are implementing those frameworks as to how it lands and how it feels for staff. **Because you may get one really positive experience in one team, but one really negative experience in another** [Trust 1, Interviewee 6, Clinical team]

This reliance on individual style and ‘vision’ of those leading that process was commonly remarked upon by participants, highlighted as a challenge given the variability in knowledge, skills and training between different investigators and safety teams. This meant that the quality and nature of an investigation was typically determined by the lead investigator and could either benefit from or be subject to weaknesses in skills, level of experience, and personal interest in engaging with patients and families. Participants described how the qualities of the lead investigator shaped the entire investigation process, and the quality and effectiveness of investigation was highly reliant on those individual traits:

Think it's very reliant on the investigator taking the terms of reference, liaising with the head of service or the service manager, identifying through the case notes review who has been involved in that particular incident, and then reaching out and having those conversations. The investigators are also the ones that will determine the relevance as well of the contact and whether they need to speak to that person in more detail. [Trust 2, Interviewee 8, Patient safety team]

This reliance on individual skill and competence was raised by participants as particularly problematic given the limited amount of training that was typically available to investigators and the lack of in-depth expertise of some of the staff involved in conducting investigations. This was complicated by certain types of particularly serious or challenging incidents needing the dedicated oversight of senior staff, who were already juggling demanding workloads, and occasionally resulted in investigations being conducted by staff who lacked appropriate training:

We have people being assigned to do investigations who haven’t ever done one before and they're just sort of nominated to do it and then it, you know, and they haven't got training. [Trust 6, Interviewee 4, Patient safety team]

The challenges of these individual variabilities in competence and skill were addressed, where possible, by managers working to allocate investigators with certain competencies to the most relevant investigations – for example, if a particular incident was expected to involve highly sensitive and complex interactions with family members:

And I think a lot of it is the confidence of the practitioners that are undertaking the reviews […] if I had one incident where I knew particularly that there was a lot of failings in care, that the family were going to be very aggrieved in terms of the process, and it was gonna be a really awful Coroner's experience for anybody involved **I'd put my best investigator on that case who I knew could really engage with families**. [Trust 1, Interviewee 1, Senior Management team]

However, given the capacity constraints described previously, and the variability in skills and training, this could be challenging and indicates the degree to which the delivery of the SIF policy aspirations of learning, engagement and improvement were constrained by and reliant on the individuals within investigation roles and their variable skills.

#### 4.3.2 Relational inconsistency and fragility

Investigative processes necessarily involve working with a range of staff, teams, stakeholders and, at time, different organisations. Likewise, following up action plans and making changes in response to investigations depend on communicating and interacting with staff in different teams and at different levels of an organisation. Participants described that it was often a challenge to engage with and develop robust and effective relations with the full range of staff and stakeholders. Concerns were highlighted that the knowledge and insight generated through investigation processes could be limited to a relatively small group of more senior staff, who are involved in safety governance processes, but that frontline staff are less reliably involved in these processes or informed of outcomes:

…as soon as I came in, I said, what about your junior members of staff? All these senior people go to safety panel and then they go to these clinical governance meetings or business unit meetings where they discuss operational stuff. They'll discuss sometimes complaints and things like that. **But your real people who are involved day to day in everything, where do they get to find out?** [Trust 3, Interviewee 9, Governance team]

It was thus challenging to build and maintain effective relations with a wide range of relevant staff and stakeholders, particularly when investigative activities are process-oriented and focus predominantly on meeting governance requirements and performance targets, rather than actively engaging with the practical work improvement and participatory learning. Similarly, some participants indicated that the need to maintain impartiality and objectivity throughout an investigative process could complicate efforts to engage with and involve staff through the process. That is, the “need to be objective, the need to have nothing to do with the incident” (Trust 5, Interviewer 5, Governance team) to ensure transparency could create difficulties in capturing staff experiences, and at times resulted in people involved in incidents being excluded from investigative processes.

As such, relations with those affected by an incident – both staff and patients/families – were not always consistent and reliable and could be distorted by efforts to comply with aspects of the SIF. Moreover, participants described how there could be a disconnect between the senior decision makers who took on responsibility to direct and determine that an incident had met the SIF criteria and would therefore require investigation, and the safety team and investigators who would then conduct those investigations:

It sat very separate if I'm honest. I think the 72-hour review document allowed team managers and clinical leads at that team-level to identify their own learning and then implement their own actions. If the decision was taken that it would go to a further investigation there was a real disconnect because that sat with the patient safety team doing the investigation and then it kind of went into the leadership team. [Trust 1, Interviewee 6, Clinical team]

Participants reported that, where collaboration between teams and across different levels of the organisation was more regularly achieved, this was often due to the development of personal and long-standing relations between key individuals, that could be developed due to the longevity of key staff holding the same position for several years. These personal relations were described as key, though were fragile and could be disrupted by a lack of continuity amongst teams – especially patient safety teams and senior managers, leadership teams, directorates, and Trust Boards. Again, this indicates that the policy aspirations and objectives of the SIF were primarily reliant on individualised and locally emergent relationships that were fragile and open to distortion and disruption, rather than clearly articulated and robustly implementing reliable systems of investigation, learning and improvement.

## 5 Discussion: How safety policies can distort safety practices and disrupt learning

**Fig. 2:**
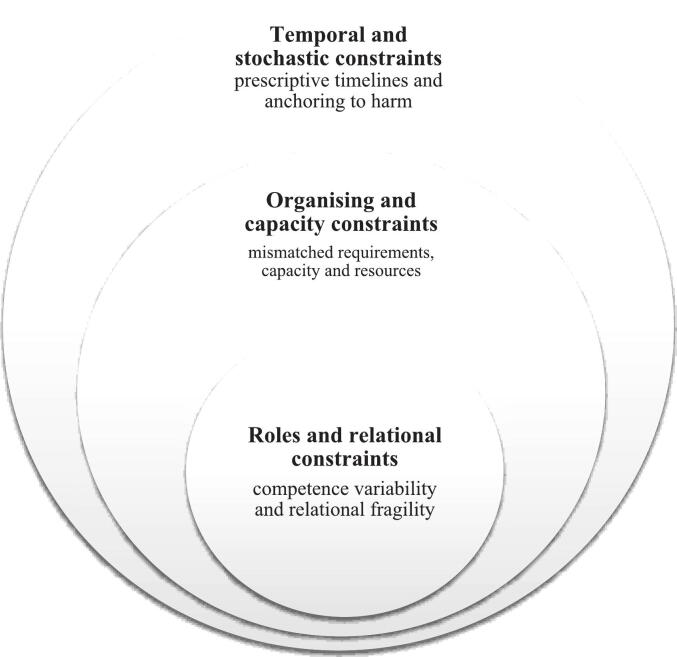
Structural, organisational and relational constraints on organisational learning identified in the interview data.

This study set out to explore the puzzle of why a national safety policy, which was intended to support rigorous investigation and learning from serious incidents, had the effect of constraining and distorting those activities in ways that hampered the achievement of the policy’s goals. The analysis developed here indicates that the SIF, the national policy which set out the framework for the investigation of serious safety incidents in the NHS between 2010 and 2023, shaped and constrained investigative practice in several unintended ways. The SIF was founded on a rigid set of timelines and outcome-driven triggers which created a temporal structure that constrained opportunities for systemic investigation, diagnosis and learning. The policy created organisational constraints in the form of challenging mismatches between organisational capacity and resources and requirements to investigate, focusing attention on policy compliance over careful exploration and comprehension of safety risks. The policy also shaped roles and relations around incident investigation, providing little guidance on the knowledge, skills, roles and resources required to manage and deliver effective investigations, or the collaborative and participative work that is generally necessary to bring about genuine learning and improvement. These nested layers of constraints amplified and reinforced each other (Fig. 2).

### 5.1 Temporal structures and learning: Deadlines over diagnosis

While the SIF promulgated a range of worthy principles intended to support open, collaborative and systemic learning, at the core of the policy was a rigid temporal structure: timelines that determined investigative reporting deadlines, notably the 60-day limit for ‘closing’ investigations. While these deadlines were intended to ensure timely responses to serious events, they necessarily created considerable time pressure. Moreover, the trigger SI investigations was primarily tied to the severity of the harmful or damaging outcomes. This was an uncertain and unpredictable trigger: the severity of incident outcomes in complex systems is necessarily stochastic and not always directly related to the extent or seriousness of underlying organisational problems. The severity of incident outcomes has long been regarded as a poor indicator of a system’s underlying safety (Reason, 1997). Put another way, “Chance does not take sides. It afflicts the deserving and preserves the unworthy” (Reason, 2000, p. 5). By imposing both a strict timeline for the completion of investigations and primarily tying the trigger for investigations to the severity of an incident’s outcome, the SIF create a temporal structure that was not supportive of extensive, exploratory and systemic investigation while also anchoring this constant tempo of investigative activity to the occurrence of harmful outcomes, rather than a strategic approach to systemic risks. The temporal structuring of organisational life can be a powerful constraining force on organisational practices (Orlikowski & Yates, 2002), and the SIF combined an event-based structure with a deadline-mandating, clock-based structure which served to both constrain the flexibility and agency of organisations in determining what to investigate, as well as consuming and committing organisational resources in when to conduct those investigations. This constraining temporal structure, along with its rapid and unpredictable rhythm, was the dominant experience of the SIF, and ensured that organisational attention became heavily focused on investigative deadlines, to the detriment of conducting effective organisational diagnosis.

### 5.2 Organisational processes and learning: Compliance over comprehension

The imposed structural requirements had important organisational consequences, particularly for the ability and capacity of organisations to meet the investigative requirements of the SIF, and secondarily in terms of the engagement and involvement of key stakeholders in these processes – particularly patients and families. The requirements for investigating serious incidents that met the SIF criteria created challenges for organisations and generated a commonly experienced mismatch between resources and policy requirements. Where organisations faced resource constraints, and were also subject to heavy regulatory scrutiny and external monitoring, organisational attention and capacity focused on meeting investigative deadlines – on ‘closing’ investigations. The SIF policy, and the regulatory environment it was implemented within, therefore created conditions that were particularly conducive of ‘secondary risk management’ (Power, 2007), in which organisational effort becomes focused on and organised around mitigating secondary risks (such as regulatory sanction) rather than exploring and comprehending the primary risks (such as underlying organisational problems) that threaten the safety of services and patients. Similarly, by specifying detailed policy requirements, timelines and objectives, but providing little articulation of, or support for, the organisational capacities and resources required to deliver those requirements, the SIF reproduced common conditions for policy implementation failure: neglecting the organisational realities, complexities, interdependencies and competing priorities that characterise the context which any policy is necessarily put into practice (e.g., Pressman & Wildavsky, 1984).

### 5.3 Role relations and learning: Procedures over participation

The organisational constraints created by the SIF were compounded by how investigative roles and role relations were shaped (and often undermined) by the policy and its implementation. The SIF did indicate the need for appropriately trained skilled and knowledgeable investigators. However, relatively little guidance was provided on those required competencies, how investigative roles should be created, maintained and resourced within organisations, or the organisational structures and support that should be put in place to enable effective and expert investigative practice and professional development. There is a subtle irony here, in that one of the most prominent issues that is often identified in safety incident analyses is related to gaps in training (e.g. Peerally et al., 2024); and yet the training and expertise of healthcare safety investigators themselves was indicated to be variable and sometimes lacking. Importantly, the constraints imposed by the strict temporal structure, and the mismatch of organisational resources, translated into investigative activities that involved only circumscribed and limited interaction with those affected by SIs (patients, families and staff), and those who should be involved in learning from incidents and enacting change and system improvements. Complying with the SIF was broadly interpreted in terms of the reporting timelines, governance procedures and related incident management activities; it was rarely reported to involve extensive collaboration with diverse constituencies of staff to understand risks and improve systems, and it was therefore not enabling of the engaged and participative learning which patient safety incident reporting systems are capable of (Macrae, 2008, de Kam et al., 2020) – and indeed depend upon and enact in other safety–critical sectors (Macrae, 2014). This failure to build deep and participative networks around risk, and the struggles that were reported to share insights, information and lessons from investigations across professional and organisational boundaries, represents another subtle irony: it echoes the structural secrecy (Vaughan, 1996) and the epistemic barriers (Kok et al., 2022) that can hamper learning and foment future organisational crises.

## 6 Conclusion

Learning from serious incidents is one of the most prominent activities in safety management across a range of safety–critical sectors. Healthcare has long undertaken considerable efforts to build and support processes of incident reporting and safety investigation. The policies and processes through which such efforts are enacted are critical, but without careful policy design and implementation, safety policies can undercut and distort organisational efforts to investigate and learn from incidents. Rigid temporal structures and reporting timelines can prioritise efforts to close down investigations rather than open up processes of collaborative and participative learning. The challenges and constraints analysed in relation to the English health system’s SIF indicate that future efforts to support rigorous analysis of incidents and enable systematic improvement might better focus attention on supporting, governing and assessing the practical activities of participative learning around risks (Macrae, 2014, Kok et al., 2022), rather than procedures, processes and stages of incident management.

## CRediT authorship contribution statement

**Polina Mesinioti:** Writing – review & editing, Writing – original draft, Methodology, Investigation, Data curation, Conceptualization. **Carl Macrae:** Writing – review & editing, Writing – original draft, Funding acquisition, Conceptualization. **Laura Sheard:** Writing – review & editing, Methodology, Conceptualization. **Sarah Hampton:** Methodology, Investigation. **Gemma Louch:** Writing – review & editing, Project administration, Conceptualization. **Jane O’Hara:** Writing – review & editing, Funding acquisition, Conceptualization.

## Declaration of competing interest

The authors declare the following financial interests/personal relationships which may be considered as potential competing interests: Jane O’Hara and Carl Macrae reports financial support was provided by National Institute for Health and Care Research. If there are other authors, they declare that they have no known competing financial interests or personal relationships that could have appeared to influence the work reported in this paper.

## Data Availability

The data that has been used is confidential.

## Footnotes

1 https://responsestudy.leeds.ac.uk/.
